# Neuroprotective Effect of* Fagopyrum dibotrys* Extract against Alzheimer's Disease

**DOI:** 10.1155/2017/3294586

**Published:** 2017-04-20

**Authors:** Chen Liang, Jian-Ping Yuan, Tao Ding, Lv Yan, Lu Ling, Xin-Fu Zhou, Yue-Qin Zeng

**Affiliations:** ^1^Key Laboratory of Stem Cells and Regenerative Medicine, Institute of Molecular and Clinical Medicine, Kunming Medical University, Kunming, China; ^2^Department of Pharmacy, First People's Hospital of Qujin, Qujin, China; ^3^School of Pharmacy and Medical Sciences, Sansom Institute, University of South Australia, Adelaide, SA, Australia

## Abstract

Accumulated evidence suggests that polyphenolic antioxidants present in herbs play important roles in prevention of AD; the molecular mechanisms behind neuroprotective actions rely on the phenols through different effects on the amyloid-aggregation pathway.* Fagopyrum dibotrys* is a traditional herbal medicine which contains high quantity phenols. In present study, we investigate the beneficial pharmacological actions of* Fagopyrum dibotrys* extract in the APP/PS1 transgenic mouse mode; meanwhile, effects of the FDE on the fibrillation and cytotoxicity of A*β* peptide were evaluated in vitro. After 9-month treatment, FDE exhibited multifunctional properties on A*β*-related pathologies, which cleaned A*β* deposits in the brain and decreased A*β* burden in the plasma, inhibited microhaemorrhage, and reduced reactive microglia in APP/PS1 transgenic mice and also promoted A*β* fibrils disaggregation and inhibited neurotoxicity induced by A*β* in SH-SY5Y cells. These results highlighted that FDE is an AD type pathology modulator with therapeutic potential against AD.

## 1. Introduction

Alzheimer disease (AD) is progressive and neurodegenerative disorder disease among the elderly, characterized by progressive loss of memory and cognition [1]. Histopathological hallmarks of AD include deposition of *β*-amyloid plaques and formation of neurofibrillary tangles, intracellular neurofibrillary tangles, reactive microgliosis, and astrogliosis [2]. Although several compounds undergoing research appeared to be protective and therapeutic effects on AD model, none of these drugs was able to stop or reverse the course in patients with AD.

*Fagopyrum dibotrys *(D. Don.) Hara is an erect perennial herb with clusters of small pinkish or white flowers and edible triangular seeds, growing mainly in China, India, Vietnam, Thailand, and Nepal [3, 4]. In China, its rhizome was regarded as folk medicine for the treatment of lung diseases, inflammation, dysentery, traumatic injuries and rheumatism [5]. The* Fagopyrum dibotrys *extract (FDE) was characterized by the abundance of proanthocyanidins, hecogenin, ferulic acid, catechin, gallic acid, epicatechin, and so on. Some polyphenol compounds such as ferulic acid and epicatechin have been reported to exhibit neuroprotective properties including therapeutic action in AD [6, 7] that may be attributed to these polyphenol metabolites which are able to cross the BBB and to penetrate and accumulate in the brain at pharmacologically relevant sub-*μ*M to *μ*M concentration [8], tightly controlling the influx in the brain of metabolites and nutrients as well as of drugs [9]. The total polyphenols content in* Fagopyrum dibotrys* is about 37.7% according to the test report provided by R&D department of Qujin Gelikang Science and Technological Co. Ltd. But up till now no studies have been carried out to show the effect of total polyphenols content in* Fagopyrum dibotrys* on the modulation of A*β*-related pathologies. Here, we report that FDE can influence the A*β* aggregation and A*β*-related pathology process.

## 2. Materials and Methods

### 2.1. Preparation of* Fagopyrum dibotrys* Extract

Fresh plant materials were collected from GAP planting base of* Fagopyrum dibotrys *in Qujing city, Yunnan province of China, which were identified by expert of Kunming Institute of Botany, Chinese Academy of Sciences. The whole* Fagopyrum dibotrys *plant was washed and cut into small pieces and dried in shade. 1000 g fresh plant dried in shade yielded approximately 200 g powder. The dried powder 100 g was milled and extracted with 60% EtOH at 90°C (extract 3 times with 1 h each time) and the crude extract was eluted with 60% ethanol and then absorbed and purified by polyamide column chromatography. Ethanol eluted extract was concentrated by a rotary evaporator at 40°C and freeze dried and a standard extract of* Fagopyrum dibotrys* (36 g) was obtained.

### 2.2. HPLC Analysis of Polyphenols in FDE

The standard extract of* Fagopyrum dibotrys *was analyzed by high performance liquid column (HPLC/UV). In brief, 25 mg FDE was dissolved in ethanol-0.1% formic acid (10 : 90) to final concentration 0.5 mg/ml. An aliquot of the sample solution 10 *μ*L was automatically injected into the column for analysis of polyphenol components. The separation was performed on ODS-C18 column (100 × 4.6 mm, 3 *μ*m) with gradient elution by the mobile phase A, methanol-0.1% formic acid (10 : 90), and mobile phase B, methanol-acetonitrile-0.1% formic acid (50 : 30 : 20); the gradient elution processes is 0 min (0% B), 15 min (8% B), 45 min (30% B), 60 min (35% B), and 75 min (100% B); the flow rate was 1.0 mL/min; the column temperature was 40°C; and the detection wave length was set at 280 nm. This method was accurate in identification and determination of compounds in* Fagopyrum* extracts and most of chromatogram peaks were identified by HPLC-MS/MS.

### 2.3. A*β* Disaggregation Assay

#### 2.3.1. A*β* Preparation

Synthetic A*β*42 was purchased from American Peptide and prepared following the protocols described previously [10]. In brief, the A*β*42 peptide was dissolved in 1,1,1,3,3,3-hexafluoro-2-propanol (HFIP, Sigma) at 1 mg/ml and was aliquoted in Eppendorf tubes. The HFIP was allowed to evaporate in the fume hood, and the resulting clear peptide film was dried under vacuum overnight.

#### 2.3.2. Thioflavin T Fluorescence Assay

To test the inhibitory effect of FDE on A*β* aggregation, 1 *μ*M A*β*42 monomer was first incubated with a serial gradient of FDE in DMEM at 37°C for 10 d to form A*β* fibril; the resultants were incubated with 5 *μ*M Th T solution. Fluorescence intensity was monitored at an excitation wavelength of 450 nm and an emission wavelength of 482 nm by a spectrometer (Turner Biosystems).

#### 2.3.3. Electron Microscopy (EM) Assay

In brief, copper grids were preplaced on the bottom of wells in a 24-well plate where 1 *μ*M A*β*42 monomer was incubated with or without FDE at a concentration gradient for 7 days at 37°C. Following incubation, the mixtures were stained with 2% (wt/vol) aqueous phosphotungstic acid for 30 min. The images were collected using a Hitachi7650 TEM equipped with Mega view 3 Digital Camera.

#### 2.3.4. SH-SY5Y Cell Culture and Viability Assay

Human neuroblastoma cell line SH-SY5Y was cultured in RPMI-1640 supplemented with 10% FBS at 37°C. Cells at 60–70% confluence were treated with 1 *μ*M A*β* with or without FDE (2.5 mg/mL, 5 mg/mL, or 10 mg/mL) for 24 hours, followed by incubation with MTT (0.5 mg/ml) for 4 h and 10% SDS solution for another 15 min at 37°C. The optical density at 560 nm was evaluated by a microplate reader (Turner Biosystems).

#### 2.3.5. Transgenic Mouse Model

APP/PS1 transgenic mice were provided by the South model animal institute, Shanghai. These mice were constructed on a C57BL/6 background and bear a chimeric mouse/human (Mo/Hu) APP695 with mutations linked to familial AD (KM 593/594 NL) and human PS1 carrying the exon-9-deleted variant associated with familial AD (PS1dE9) in one locus under control of a brain- and neuron-specific murine Thy-1 promoter element [11]. Genotypes of the off spring were determined by PCR analysis of tail DNA. Mice were maintained on ad libitum food and water with a 12 h light/dark cycle.

#### 2.3.6. Diet Treatment

The transgenic mice were randomly divided into 3 groups, each group comprising 12 mice, male : female = 1 : 1. The FDE (Tg) group were treated with 0.65% FDE mixed food; the dosage (0.103 mg/kg/d) used in the current study was based on clinical data. Control (Tg) and another age and sex matched wild-type mice group were fed with standard commercial food (Beijing Keao Xieli Feed Company, Beijing, China). From 3 months old, all the mice were fed the above diets for another 9 months. Food consumption and animal body weight were detected every 3 months during treatment period.

In this study, all experiments were performed in accordance with the European Communities Council Directive (2010/63/UE) and NIH for the Care and Use of Laboratory Animals. The study had been approved by the Committee for Animal Experiments and Ethics at the Kunming Medical University.

## 3. AD Type Pathology and Bioanalysis

### 3.1. Tissue Sampling

After 9 months of treatment, the mice were anesthetized with 10% chloral hydrate and the blood was collected from the eyeball for biochemical detection. Next, the brain was perfused with 50 ml chilled standard saline solution and was removed and bisected in the mid-sagittal plane. The left brain hemisphere was fixed in 4% paraformaldehyde (pH 7.4) for 24 h and incubated for another 48 h in 30% sucrose for subsequent cryoprotection. Coronal sections of the brain were cut at 35 *μ*m thickness d with a cryosectioning microtome and stored at 4°C in PBS containing 40% glycol until use. The right brain was snap frozen in liquid nitrogen and stored at −80°C for future biochemical analysis.

### 3.2. Pathological Staining and Image Analysis

A series of five equally spaced tissue sections (~1.3 mm apart) were randomly selected and stained using free-floating immunohistochemistry (Biotin-conjugated mouse anti-A*β* antibody 6E10, Serotec) and Congo red for total A*β*, activated microglia (rat monoclonal anti-CD45 Chemicon, USA), and astrocyte (rabbit polyclonal antiglial fibrillary acidic protein, Dako, Denmark), respectively. Sections were incubated overnight with primary antibodies at 4°C, further developed with biotinylated secondary antibodies, and visualized with diaminobenzidine (Slide Kit Chemical international, Inc. Millipore).

For the compact A*β* plaque staining, a series of sections was mounted and stained with Congo red. In brief, the sections were treated with working sodium chloride solution (containing sodium chloride saturated in 80% alcohol and 0.01% sodium hydroxide) at room temperature for 30 min, then placed directly into working Congo red solution (containing saturated Congo red in working sodium chloride solution) for 1 h, and dehydrated rapidly in absolute alcohol.

The region of neocortex and hippocampus manually was selected for quantification of total A*β* plaques and microgliosis. The images were acquired in the same session and collected at 4x magnification using constant bulb temperature and exposure (Olympus DP73), yielding the area fraction of the total positive staining against the area of tissue analyzed; the average number of deposits was calculated per each brain area; all image analyses were developed in a blind manner.

### 3.3. Quantification of A*β* Peptide Levels by ELISA

ELISA analysis of the brain A*β* was processed as described previously [12]. Briefly, frozen brain was homogenized and sonicated in TBS containing protease inhibitors. Homogenates were centrifuged at 100,000 ×*g* for 1 h at 4°C, and the resultant supernatant was collected, representing the TBS-soluble fraction (A*β*-TBS). The resultant pellet was suspended and sonicated in water containing 2% SDS and protease inhibitors. The SDS solubilized homogenates were centrifuged at 100,000 ×*g* for 1 h at 4°C, and the resultant supernatant was collected, representing the SDS-soluble fraction (A*β*-SDS). The resultant pellet was then extracted in 70% formic acid (FA) and centrifuged, and the resultant supernatant was collected, representing the SDS-insoluble fraction (A*β*-FA). Before ELISA assay, formic acid extracts were neutralized by 1 : 20 dilution into 1 M Tris phosphate buffer, pH 11, and then diluted in sample buffer. Concentrations of A*β*40 and of A*β*42 in brain extract and serum were quantitatively measured by ELISA following the manufacturer's instructions (ELISA kits, Millipore).

### 3.4. Quantification of IL-1*β*, TNF-*α*, and IFN-*γ* in the Mouse Plasma by ELISA

IL-1*β*, TNF-*α*, and IFN-*γ* in the plasma of mice were measured using ELISA kits as per manufacturer's instructions (ELISA kits, eBioscience).

### 3.5. Assessment of Toxicity of FDE

Total bilirubin, alanine aminotransferase (ALT), and aspartate aminotransferase (AST) were analyzed by Analytical Biochemical Laboratory of First Affiliated Hospital of Kunming Medical University.

## 4. Statistical Analysis

Results were generally presented as mean ± standard error of the mean (SEM). Statistical analysis was performed using one-way ANOVA followed by post hoc Student's test where appropriate; *p* < 0.05 were considered statistically significant. Statistical analysis was performed using SPSS for Windows version 20.0 (SPSS Inc.).

## 5. Results

### 5.1. HPLC Analysis of Polyphenol Compounds in FDE

**Figure 1:**
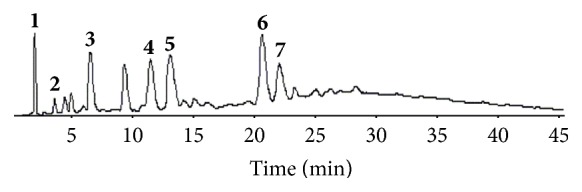
The HPLC/UV chromatograms of* Fagopyrum dibotrys* extract. Peaks:** 1.** gallic acid;** 2.** protocatechuic acid;** 3**. catechin;** 4.** dimer gallic acid ester;** 5** epicatechin;** 6.** trimer gallic acid ester;** 7**. epicatechin gallate.

The presence of polyphenols in the extracts was confirmed by identifying characteristic spectral features and comparison with standard compounds (>95% purity by HPLC, Shanghai Tauto Biotech Co., Ltd.). The use of reversed phase high performance liquid chromatography allowed the identification of 7 phenolic constituents; there were gallic acid, protocatechin acid, catechin, dimer gallic acid ester and epicatechin, trimer gallic acid ester, and epicatechin gallate (Figure 1).

### 5.2. Effects of the FDE on the Fibrillation and Cytotoxicity of A*β* Peptide

**Figure 2:**
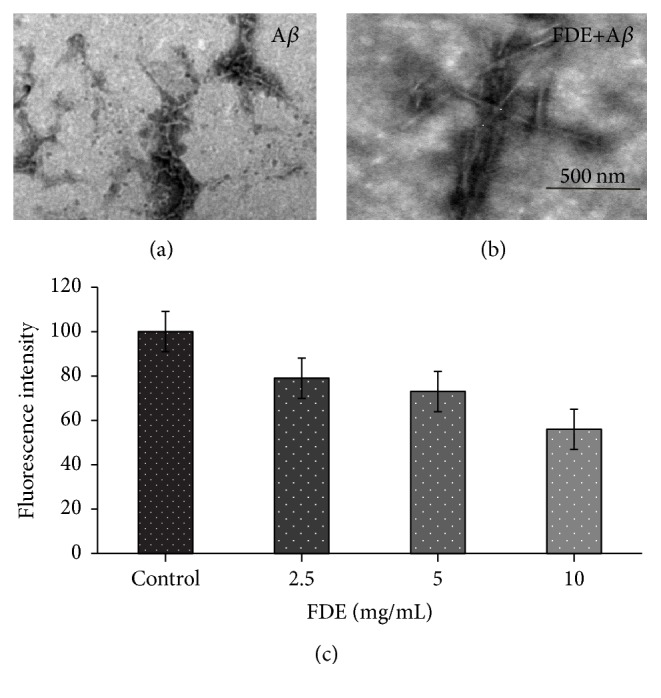
FDE disaggregate of A*β* fibrils. (a) Preformed A*β* 1 *μ*M was incubated alone at 37°C 5 d. (b) A*β* with FDE at 37°C for an additional 3 d. Scale bar, 500 nm. (c) Th.T assays for effect of FDE on disaggregation of preformed A*β* fibrils (*n* = 3, *p* < 0.05).

**Figure 3:**
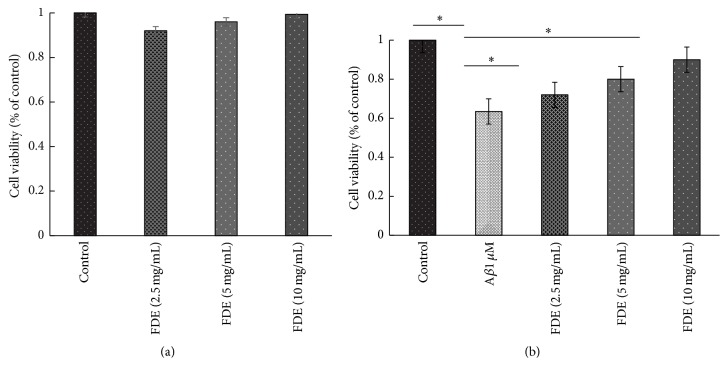
FDE attenuates the toxic effect of A*β*42 in SH-SY5Y cells. MTT assay of cell viability of SH-SY5Y cells treated with (a) various doses of FDE for 24 h (^*∗*^*p* > 0.05) and (b) 1 *μ*M A*β*42 with various doses of FDE (^*∗*^*p* < 0.05).

To investigate the effects of FDE on the fibrillation and cytotoxicity of A*β* protein, the inhibitory effects of FDE on A*β*1–42 fibril formation were determined by using thioflavin T fluorescence (Th-T) and Electron Microscopy (EM); the protective effects against cytotoxicity induced by A*β*1–42 in SH-SY5Y cells were evaluated by MTT assay. The data from Th-T fluorescence assay showed that FDE had a dose-dependent effect on disaggregation of preformed A*β*1–42 fibril (ANOVA, *F* = 10.762, *p* < 0.001, Figure 2(c)), it also reduced the neurotoxicity of A*β*1–42 on the cultured SH-SY5Y cells (Figures 3(a) and 3(b), *p* < 0.05).

The morphologies of the assemblies present following A*β* incubations with or without FDE were examined using Electron Microscopy (EM). When A*β*42 was incubated under fibril-forming conditions, different morphology of amyloid fibrils such as nonbranching fibrils, twisted long fibrils, and some small aggregates mixed with fibrils could be observed (Figure 2(a)). Conversely, when preformed A*β* fibrils were further incubated with FDE for an additional 3 days (Figure 2(b)), there were hardly any long fibrils on squares of grids.

These data indicate that FDE can effectively inhibit A*β*1–42 fibril formation and significantly lower the neurotoxicity on the cultured SH-SY5Y cells, suggesting potential effect of the FDE on A*β* clearance.

### 5.3. FDE Is Well Tolerated in APP/PS1 Transgenic Mice

**Figure 4:**
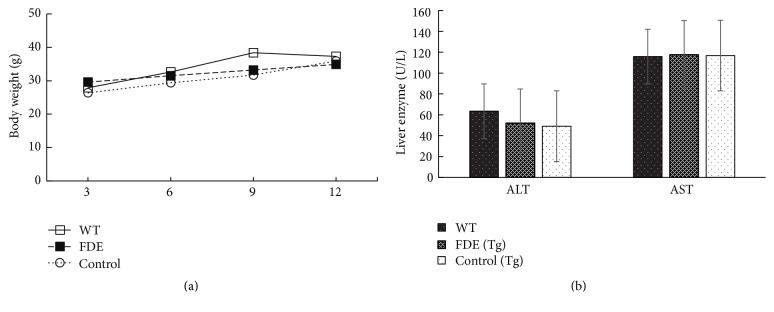
FDE is well tolerated in APP/PS1 transgenic mice. (a) Body weight was monitored at 3, 6, 9, and 12 months of age. (b) Serum levels of ALT and AST (*p* > 0.05). Points and bar graphs represent group mean (±SEM).

During treatment period, we did not observe any animal death occurring and any differences in animal viability general activities among the groups. It is important to note that the long-term daily consumption FDE diet for 9 months in APP/PS1 transgenic mice did not significantly influence animal body weight and liver function (Figures 4(a) and 4(b), *p* > 0.05, the serum level was too low to be detected). The FDE was well tolerated in AD mice.

### 5.4. FDE Attenuates A*β*-Related Pathologies

**Figure 5:**
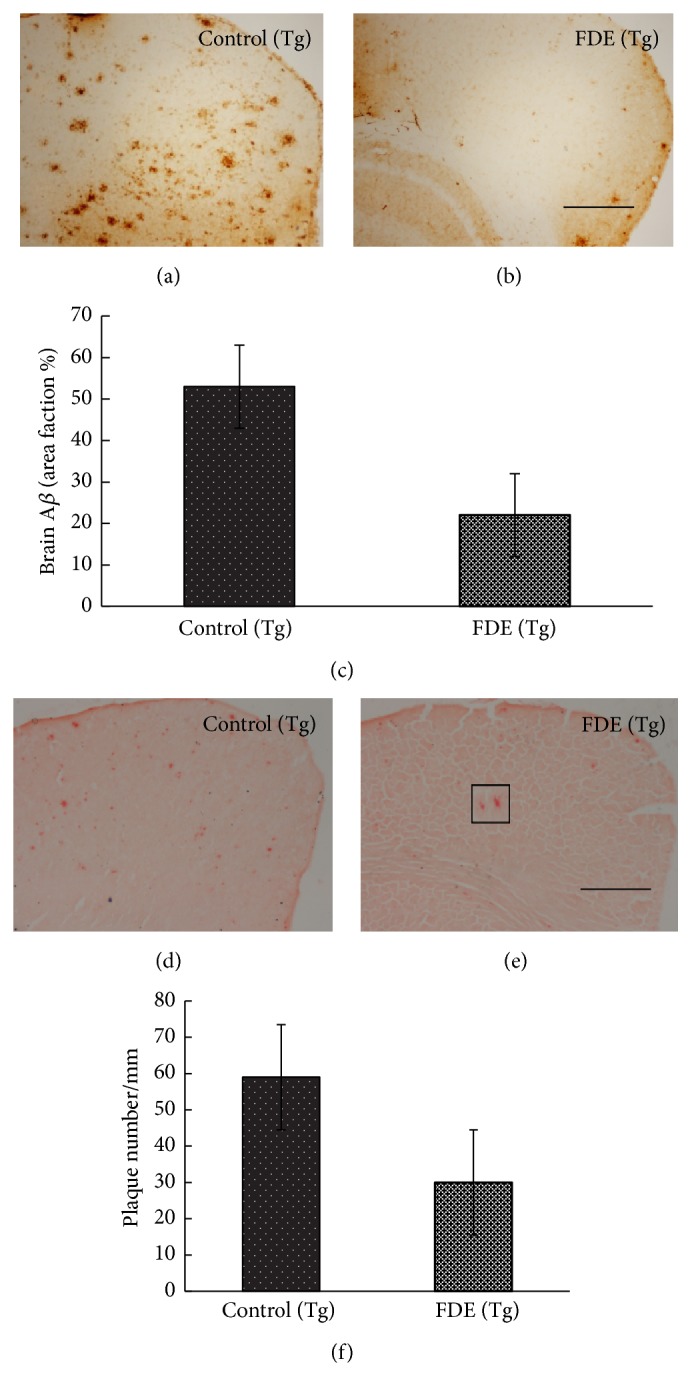
A plaque burden in the brain of mice. (a-b) IHC-positive A*β* plaques in neocortex of 9-month-old mice. (c) Comparison of IHC-positive A*β* plaque density in neocortex of 9-month-old mice. (d-e) Congo red-positive A*β* plaques in neocortex of 9-month-old mice. (f) Comparison of Congo red-positive A*β* plaque density in neocortex of 9-month-old animals. Denote *p* < 0.05 versus control group.

In order to know A*β* deposition in the brain after FDE treatment, the AD brain sections were stained with anti-A*β* antibody 6E10 and Gongo red; a histological dye is commonly used to detect fibrillar A*β* plaques [13]. A*β* plaques were observed primarily in the neocortical and hippocampal areas of the brain, as observed in Figures 5(a), 5(b), 5(d), and 5(e).

**Figure 6:**
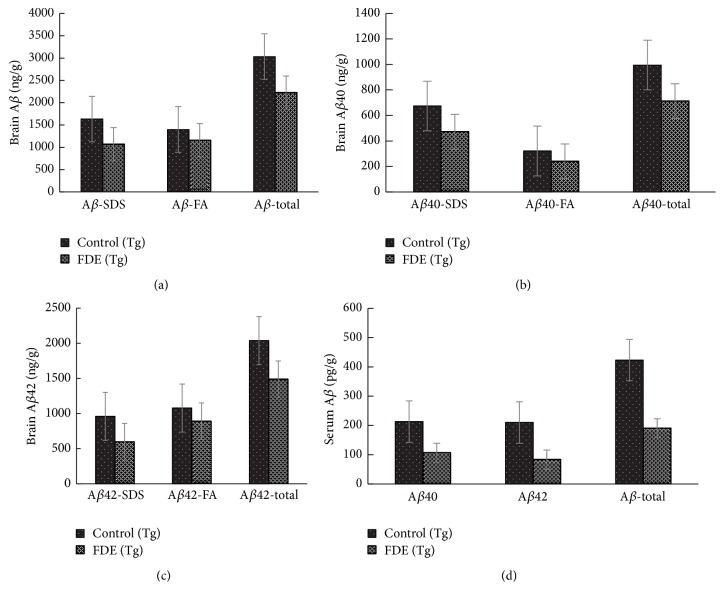
Effects of FDE consumption on A*β* levels in the brain and serum of APP/PS1 transgenic mice. (a) Comparison of total A*β*, A*β* in SDS fraction (A*β*-SDS), and A*β* in formic acid fraction (A*β*-FA) among groups. (b) Comparison of total A*β*40, A*β*40-SDS, and A*β* 40-FA. (c) Comparison of total and A*β*42-FA. (d) Comparison of total, A*β*42, A*β*42-SDS A*β* 40, and A*β* 42 in serum. Denote *p* < 0.05 or *p* < 0.01 versus APP/PS1 transgenic mice fed with control diet. Scale bar, 1 mm.

The total number of A*β* plaques stained by immunohistochemistry and Congo red in the brain was, respectively, reduced by 57% and 49% (Figures 5(c) and 5(f)); FDE treatment group had a significantly lower A*β* plaques numbers than control group fed with normal diet (ANOVA, *F* = 5.420, *p* < 0.05, and ANOVA, *F* = 6.480, *p* < 0.05). We further examined the brain and plasma A*β* levels by ELISA tests; the levels of total A*β*40 and A*β*42 in SDS and FA fractions of brain homogenates of FDE treatment group were significantly declined compared to control group (Tg) (ANOVA, *F* = 71.044, *p* < 0.05; ANOVA, *F* = 15.481, *p* < 0.001; ANOVA, *F* = 8.425, *p* < 0.05); the levels of A*β*40 and A*β*42 in formic acid (FA) also showed significant decline (Figures 6(b) and 6(c), *p* < 0.05). In addition, the total A*β* level in plasma of FDE treatment group obviously reduced by 55% (Figure 6(d)), which correlated with the change tendency of total brain A*β* levels (Pearson *r* = 0.883, *p* < 0.05).

**Figure 7:**
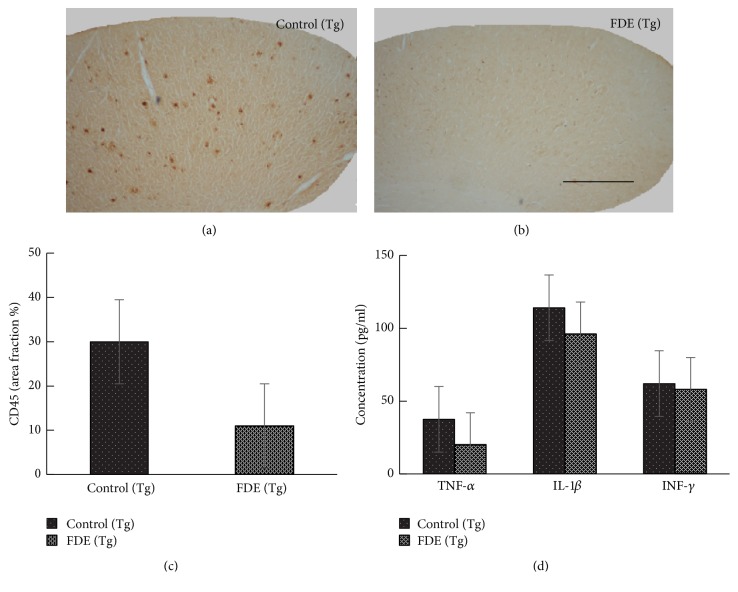
FDE attenuates inflammation in APP/PS1 transgenic mice. (a–c) Representative images of staining in APP/PS1. No obvious microgliosis was observed in the brain of FDE treatment transgenic mice. (c) Comparison of CD45 area fraction in neocortex among groups. (d) Quantification of IL-1*β*, IL-6, TNF-*α*, and IFN-*γ* in plasma. Denote *p* < 0.05 or *p* < 0.01 versus control (Tg) mice, as determined by one-way ANOVA. Scale bar, 1 mm.

**Figure 8:**
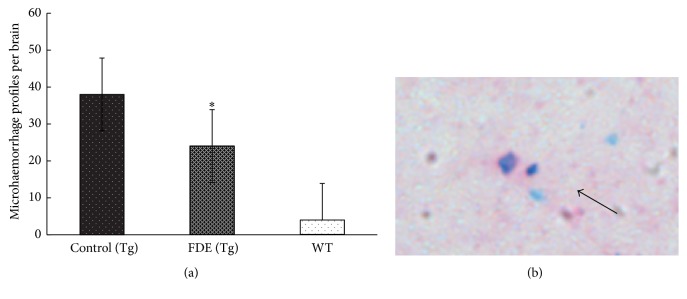
Effects of FDE treatment on microhaemorrhage profiles in the brain. (a) Comparison of microhaemorrhage profiles; denote ^*∗*^*p* < 0.05 versus wild-type littermate fed with control diet. (b) An example of microhaemorrhage profile (solid arrow) observed in neocortex.

Activated microglia are associated with the progression of Alzheimer's disease (AD); therefore microglia are key targets for therapeutic intervention [14]. Here, we examined the activated microglia with rat monoclonal anti-CD45 in the neocortical and hippocampal areas of the brain; the levels of activated microglia in FDE treatment group were obviously decreased compared to the transgenic mice fed with normal diet (11 ± 3 versus 30 ± 4.5, *p* < 0.001, Figure 7(c)). Moreover, we measured the levels of IL-1*β*, TNF-*α*, and IFN-*γ* in the plasma; ELISA assay showed that the level of proinflammatory cytokine TNF-*α* in serum in the FDE treatment group was lower than APP/PS1 control group (36.7 ± 4.3 versus 20 ± 2.7, *p* < 0.05, Figure 7(d)), but no significantly lower levels of IL-*β* and IFN-*γ* were observed in FDE treatment group. In Addition, characteristic blue haemosiderin-positive profiles on the stained brain slices were observed. The microhaemorrhage rates in the FDE group were detected at a rate of 24.5 ± 3.6 per hemibrain (*p* < 0.05) which was lower than Control group fed with normal diet (38.0 ± 6 per hemi-brain, *p* < 0.05, Figure 8).

## 6. Discussion

The present results showed the neuroprotective ability of FDE on AD-like symptoms in transgenic mice. Besides, the antiamyloidogenic effects of* FDE *in vitro were detected in this study. To our knowledge, this is the first time to report that polyphenol extract of* Fagopyrum dibotrys *has preventive effects on an animal model of AD pathology.

Our data demonstrate that FDE cleared A*β* deposits that appeared in the brain and decreased A*β* from the plasma, reduced microhaemorrhage, decreased reactive microglia in brain, and reduced the TNF-*α* level in serum. But, we did not observe FDE reverse deficits in motor function and spatial working memory in the Morris water maze test (result is not shown).

Other putative agents tested for enhancement of cognition in animal models can improve memory. For example, blueberry appears to have a pronounced effect on short term memory and has been shown to improve long-term reference memory following 7 weeks of supplementation [15]; the flavonoid-rich plant extract of* Ginkgo biloba *has also been shown to induce positive effects on memory, learning, and concentration [16]. The differences in effects on memory between these results and those in the present manuscript may be attributed to different strains of mice tested or different chemical structures of the compounds tested. Hence, the beneficial effects and mechanisms of polyphenols on memory deficits caused by A*β* need further research [17].

Amyloid *β* (A*β*) is toxic to neurons and is associated with multiple pathogenic cascades. Inhibition of A*β* aggregation or disaggregation of A*β* fibrils are important strategies for prevention and treatment of AD. In this study, we found that FDE significantly attenuated A*β*42 fibril-induced toxicity on SH-SY5Y cells, inhibited A*β*1–42 fibril formation in all tested concentrations, consistent with the effects of other polyphenols contained in grape seed extract, wine, bilberry, and blackcurrant, which have been shown to destabilize the preformed A*β* fibrils in vitro, and exhibited antiamyloidogenic activity [18].

New research suggests that pathological accumulation of A*β* is a key factor that drives neuroinflammatory responses in Alzheimer's disease; these A*β* aggregates bind to cell-surface receptors on microglia, inducing an inflammatory activation that results in the secretion of proinflammatory cytokines [19], including TNF-*α*, interleukin 1*β*, and IFN-*γ*. Overexpression of inflammatory cytokines has been shown to increase tau pathology [20]. Our data showed the plasma level of TNF-*α* and reactive microglia are decreased after FDE treatment, indicating that the FDE can modulate neuroinflammation and alleviate harmful effects formed under pathological conditions. Cerebral amyloid angiopathy (CAA) has been recognized as one of the morphologic hallmarks of Alzheimer disease (AD), resulting from deposition of *β*-amyloid in the media and adventitia of small arteries and capillaries of the leptomeninges and cerebral cortex, which is associated with cerebral microbleeds and white matter hyperintensities [21]. The present study found that FDE is beneficial in ameliorating cerebral microbleeds, possibly a result of clearance of the 12 amyloid-*β* peptide from the brain. This finding may suggest FDE's potential as preventive agent for cerebral amyloid angiopathy (CAA).

In conclusion, the present study showed that the oral administration of FDE prevented the development of AD pathology in an animal model by targeting multiple pathways. It provides a novel suggestion that FDE might show promise in the prevention or treatment of AD.
